# Propranolol for Treatment of Infantile Hemangioma: Efficacy and Effect on Pediatric Growth and Development

**DOI:** 10.1155/2021/6669383

**Published:** 2021-04-07

**Authors:** Rachel A. Giese, Merit Turner, Mario Cleves, J. Reed Gardner, Gresham T. Richter

**Affiliations:** ^1^Department of Surgery, Division of Otolaryngology-Head and Neck Surgery, University of Texas Rio Grande Valley School of Medicine, 1201 W University Dr, Edinburg, TX, Canada 78539; ^2^Department of Otolaryngology-Head and Neck Surgery, University of Arkansas of the Medical Sciences, 4301 W, St Little Rock, Markham, AR, Canada 72205; ^3^Morsani College of Medicine, Department of Pediatrics, Health Informatics Institute, University of South Florida, 3650 Spectrum Blvd, Tampa, FL 33612, USA

## Abstract

**Purpose:**

Propranolol has been successful in treating problematic infantile hemangiomas (IH) but concerns regarding its effect on normal growth and development have been raised. This study examines physical growth, developmental milestones, and human growth hormone (hGH) levels in infants receiving propranolol for problematic IH.

**Method:**

Monthly heights and weights of children undergoing propranolol therapy for IH were prospectively collected and tabulated. Data analysis and comparison to World Health Organization (WHO) weight-for-age and weight-to-length *z*-scores was performed. Questionnaires regarding milestones, efficacy, and guardian satisfaction were performed, and a combination of both chart results and phone conducted surveys was tabulated. Serum from a small representative cohort of age-matched children with IH treated and not treated with propranolol was collected.

**Results:**

A total of 185 children receiving propranolol therapy between 2008 and 2013 for IH were assigned to this study. The children were divided into two cohorts based on the presence of comorbidities or risk factors that may affect growth and development (*n* = 142 no comorbidities, *n* = 43 with comorbidities). Neither cohort demonstrated deviation from normal weight in comparison to WHO normative data. There was a significant deviation for BMI-for-age and weight-for-age *z*-scores in our population, especially in patients on propranolol for more than 7 months. Based on data from participants, via either completed questionnaires or chart results, most children met their developmental milestones at or before target ages, regardless of the presence of comorbidities. Eighty percent of guardians noticed clinical improvement of the IH, with 91% either happy about or neutral to using the medication. hGH levels were higher in patients receiving propranolol therapy, but not significantly different.

**Conclusion:**

Propranolol therapy is effective and well tolerated in the treatment of infantile hemangiomas. This study suggests that propranolol does not impair growth and has no impact on normal pediatric development.

## 1. Introduction

Infantile hemangiomas (IH) are benign tumors of blood vessels that, in general, start growing by the third month of life, undergo a rapid growth phase for 6-8 months, and subsequently involute at a variable rate. They are the most common benign tumor of infancy, occurring in about 5% of infants [1]. Most of them do not require treatment, but about 12% are problematic and prompt referral to a specialist and require treatment [2]. IH characteristics that stimulate referral may include airway compromise, cosmetic deformity, ulceration, bleeding, or functional loss, such as visual impairment or dysphagia. In the past, steroids, chemotherapy, laser therapy, and surgery treatments were used for problematic IH. These treatment options are becoming less necessary after the serendipitous discovery by Leaute-Labreze et al. [3] of early IH involution in a child treated with propranolol for cardiomyopathy. Since that discovery, physicians have embraced this therapy and are increasingly showing its success and safety.

Propranolol is a nonselective beta-blocker with a relatively low side effect profile, especially in healthy infants [4]. The most common side effects are sleep disturbance, dyspnea, nausea, somnolence, bradycardia, hypotension, hypoglycemia, and gastroesophageal reflux [5, 6]. However, children treated with propranolol for IH are typically asymptomatic or experience mild hypotension or bradycardia [1]. Additionally, children with preexisting hypoglycemia have been safely treated for infantile hemangiomas with propranolol [7]. Reported deaths and instances of heart failure associated with use of propranolol have been due to intravenous propranolol or overdose [8]. Following FDA approval for the treatment of IHs and its increasing use in institutions around the world [3, 5, 9–17], the American Society of Pediatric Otolaryngology Vascular Anomalies Task Force approved its use [1, 2]. Consensus conferences, international multicenter studies (NCT01056341), and meta-analysis have all shown the safety and efficacy of propranolol [18], especially in comparison to steroids [19]. Erbay et al. [20] demonstrated safety and efficacy in preterm and low birth weight infants. Some centers are successfully using a nonselective topical beta-blocker, such as timolol, with or without oral systemic propranolol to limit systemic toxicity [21, 22].

Although propranolol has been shown to have a clean side effect profile, questions regarding the effect of propranolol on normal growth and development have been raised among physicians treating children with IH. Pediatricians have questioned if potential long-term side effects of prolonged therapy on growth and development could be justified by the benefits for nonlife-threatening hemangiomas.

This study prospectively and longitudinally examines 1^st^ year growth parameters (height and weight) and developmental milestones up to 48 months of age in infants treated with oral propranolol on an outpatient basis. In addition, in a small cohort of treated patients, human growth hormone (hGH) levels were evaluated compared to age-matched normal controls. We hypothesize that propranolol does not influence normal pediatric growth and development. It is our hope that studies such as this will assuage these concerns and validate its use for children with problematic IH.

## 2. Methods

Institutional Review Board approval 202187 and informed consent were obtained. First, a retrospective review of prospectively collected data on heights and weights of children on propranolol was performed. These values were recorded from monthly dosing changes based on weight as recorded by the patient's PCP.

Patients were divided into two cohorts, those with comorbidities that could affect growth and development and those without such comorbidities. The most common reason a patient was determined to have a comorbidity was a gestational age less than 36 weeks. Other factors were prolonged NICU stay (>2 weeks), congenital heart disease requiring surgery (excluding patent ductus arteriosus unless also coarctation of the aorta), congenital airway anomalies, adrenal insufficiency, hypothyroidism, and failure to thrive. Diseases such as asthma, eczema, autism, ADHD, and isolated low birth weight were not considered significant to growth and development. Patients presented to our outpatient vascular anomalies clinic for follow-up after initiation of propranolol. The mean follow-up time was 7.2 months. The weight-for-age, BMI-for-age, and weight-to-length *z*-scores were calculated and compared to normative World Health Organization (WHO) values for pediatric growth. A mixed effects model was created with an unstructured covariance matrix to analyze weight, weight-for-age, weight-for-length, and BMI-for-age *z*-scores and percentiles. Random intercepts and random slopes were evaluated by testing nested models using likelihood ratio tests.

In order to determine age of milestone achievement, side effects, efficacy, other treatments, and satisfaction with the therapy up to 48 months posttreatment, guardians were surveyed retrospectively, and additional chart results were tabulated. In a representative cohort, serum from age-matched children with IH treated and untreated with propranolol was collected for the determination of human growth hormone (hGH) levels during the course of their treatment.

## 3. Results

One hundred and eighty-five patients were included in the study, 48 males and 137 females (Table 1). Multiple gestation (twins and triplets) was found in 7.6% of the population. Eighty-eight patients had heights and weights while 97 patients had weight alone. Questionnaires regarding milestones, efficacy, and guardian satisfaction were performed on 174 patients, and a combination of both chart results and phone conducted surveys were tabulated. Thirty-eight patients had hGH measured; 11 of whom were on propranolol with the remaining untreated and healthy age matched. Serum measurements were drawn only on patients in the cohort undergoing anesthesia for related or unrelated procedures. Blood draws were considered too invasive in the clinic setting for this study.

There were 142 patients without and 43 patients with comorbidities that affect growth and development (Table 1). Birth weight in the patients was significantly lower in the cohort with comorbidities (*p* < 0.0001) suggesting patients were appropriately chosen for the cohort without comorbidities. Laryngomalacia was reported in 5 patients, and cardiac anomalies were reported in 16 patients. Twenty-three patients had general anesthesia for surgery for something other than IH.

One patient whose guardian completed the questionnaire was excluded in the developmental portion due to global developmental delay, and another questionnaire was stopped short due to a cerebrovascular injury. If a parent could not recall the age at which milestones were achieved, that portion of the questionnaire was excluded. Families may not have responded to all the questions in the survey. Results reflect the total number of respondents for each question.

Of note, thirty-two (17.3%) patients required a postnatal NICU admission unrelated to IH therapy with a length of staying ranging from 2 to 98 days and a median of 12 days. Fifty-eight were on medications other than propranolol. The most common unrelated pediatric condition was gastroesophageal reflux, and accordingly, the most common other medication was a reflux medication such as an H2 blocker or a proton pump inhibitor (62 patients).

### 3.1. Anthropomorphic Measurements

**Figure 1:**
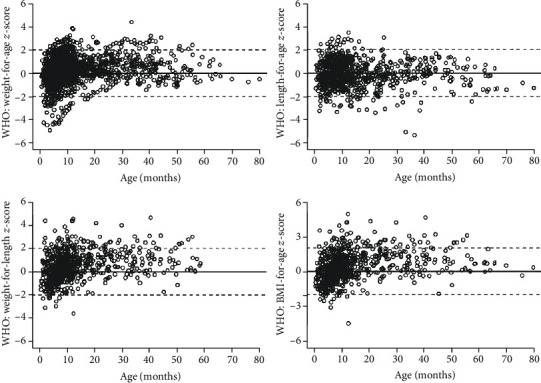
Treated and nontreated patients' *z*-scores compared to WHO standards.

**Figure 2:**
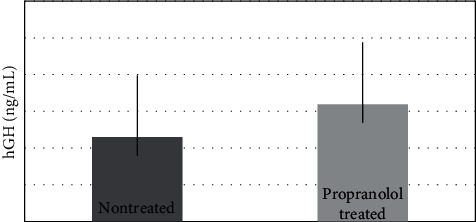
Mean hGH level in propranolol-treated (6.38 ng/mL) versus non-treated hemangioma patients (4.59 ng/mL). SD =3.15 ng/mL, *p* = 0.16.

The age-matched control heights and weights were compared in the propranolol treated and nontreated groups. The anthropomorphic data show patients in both cohorts were within or above the WHO normative values (Figure 1 and Supplementary Table 1). Some patients in our study population have higher weight-for-length and higher BMI-for-age than the WHO normative population. This observation seems to correlate with time on propranolol therapy (Supplementary Table 2). hGH levels were 6.38 ng/mL for propranolol-treated patients compared to 4.59 ng/ml in nontreated patients (Figure 2). This represents a standard deviation of 3.15 ng/ml and a *p* value of 0.16.

### 3.2. Side Effects

Twelve side effects from propranolol were reported in 70/185 (37.8%) patients but rarely led to drug cessation (Table 2). The most common side effect was reflux, found in 27 (15%) patients. Of note, 44 patients had symptomatic reflux before starting propranolol therapy, and 12 different questionnaire respondents reported it as a side effect. Other side effects included respiratory disturbances (*n* = 13), lethargy (*n* = 9), and sleep disturbance (*n* = 8).

### 3.3. Patient Satisfaction and Efficacy

Most guardian respondents reported that they were happy with the propranolol therapy 88/114 (77%); 16/114 (14%) were neutral, and 10/114 (9%) were unhappy. 152 families reported clinical (80%) improvement in color, size, or both. 107/154 (69.5%) noticed a color change and 152/174 (87.3%) reported a decrease in size of the IH, most within one month of starting therapy (60/89, 67.4%). Twelve percent of patients had rebound after propranolol was stopped, and 73.9% of patients had a second type of treatment (laser, surgery, or injection) (Table 3). Ninety-three (53.4%) patients had laser therapy in addition to the propranolol.

### 3.4. Milestones

**Figure 3:**
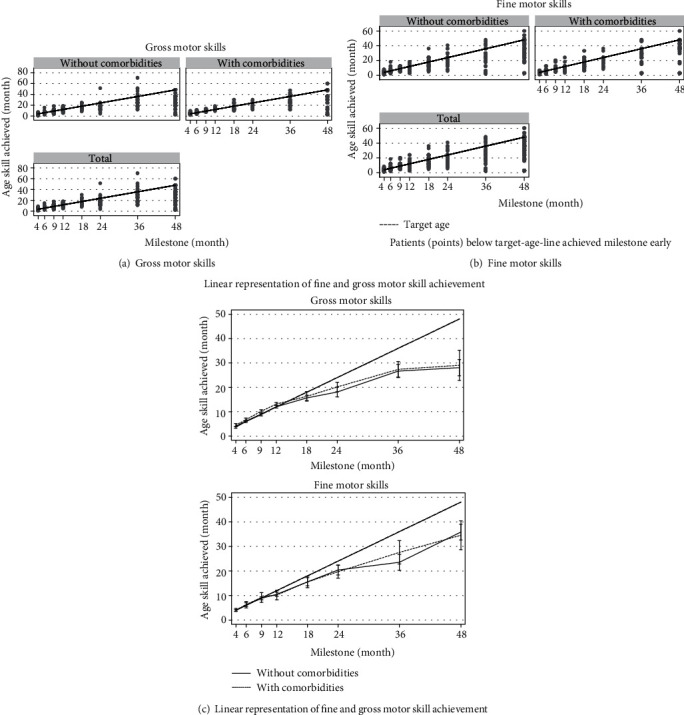
Patients treated with propranolol compared to expected developmental milestone. Each dot represents a patient at a moment in time. Patients below the target line reached the milestone before the target age.

**Figure 4:**
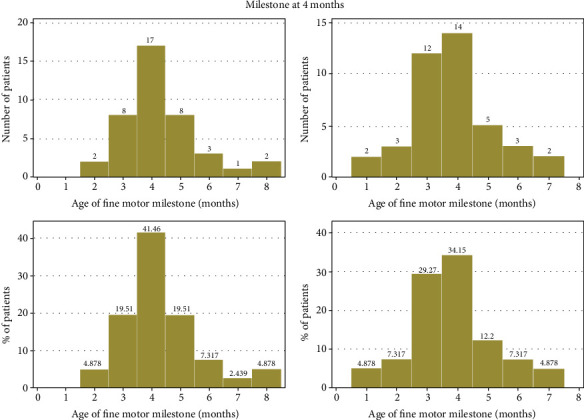
Patients treated with propranolol compared to expected developmental milestone. Number of patients per age that achieved 4-month milestone and percentage of patients per age that achieved 4-month milestone.

Based on the questionnaire respondents and chart results, patients met gross and fine motor milestones at or before the expected target age (Figure 3). This is true in both cohorts with or without comorbidities. Furthermore, by 4 years of age (Figure 4), less than 20% had not reached their expected milestone. Milestone achievement was not impacted by the presence of comorbidities that affect growth and development (Table 3).

## 4. Discussion

Propranolol has been in use for the treatment of infantile hemangiomas since 2008 and is well tolerated as evidenced in the present study. Although 12 different side effects were reported, some may have been observations and not directly related to propranolol use. Fifteen percent of our population reported reflux as a side effect. Although “spitting up” and “reflux” are difficult to distinguish, 10% of guardians thought that their children spit up more on propranolol.

In addition to being well tolerated, propranolol is known to be a successful treatment for infantile hemangiomas. In our study, most respondents were happy with the therapeutic effect. However, almost 74% of our population received secondary treatment, with the majority (53.4%) of those receiving laser therapy. Laser therapy was performed to control symptoms such as bleeding or as a cosmetic adjunct for remaining tissue rather than for a propranolol treatment failure. A flash pump dye laser at 595 nm was used for this purpose. In most cases that required surgical excision, propranolol was used preoperatively, and surgery was then performed to remove remaining anetoderma or scar revision. All indices of efficacy, satisfaction, and side effects were not different between the cohorts with and without comorbidities.

Though there is not a single human study showing developmental delay or growth retardation due to propranolol, concern might have been derived from previous studies showing intrauterine growth restriction (IUGR) and decreased perinatal growth in rats who were born to dams on beta-blockers [23, 24]. Another animal study showed propranolol caused body weight deficits up to 33% [25]. Of note, this was at a dose of 75 mg/kg lavaged into suckling rats. Based on consensus data propranolol dose used to treat IH is recommended to be 2-3 mg/kg/day [2]. Thus the lower dose of propranolol given to humans should not pose the same threat as the high dose used in rat studies. In fact, recent work by Moyakine et al. demonstrated no impact of low dose propranolol on human growth and development up to 4 years of age in infants treated for IH. The present findings refute prior animal investigations and corroborates prior human investigations.

Contrary to the concerns about pediatric growth, Ghigo et al. demonstrated beta-adrenergic antagonism increases growth hormone releasing hormone-induced GH secretion [26]. There is no known correlation between IH and growth hormone except one case of GH deficiency in a patient with facial hemangioma in the context of PHACE syndrome [27]. Accordingly, measurement of growth hormone in this study was chosen as an added objective measurement of pediatric growth in addition to heights and weights with evidence of higher hGH levels in patients treated with propranolol. Similarly, Altin et al. demonstrated that the physical growth rate of patients with a mean age of 2.64 was not affected by propranolol [28].

Because propranolol has been shown to affect the consolidation of memory [29], a concern was expressed that this may affect cognitive development in children. However, a study including 36 children with infantile hemangiomas on oral propranolol showed no effect on neurodevelopmental outcomes or CNS development [30], as well as another study in propranolol-treated children aged five to seven-and-a-half years old that showed no significant evidence of intelligence or memory development disturbance [31].

Our data suggest propranolol is a well-tolerated, effective treatment that does not impair normal growth and development. Although great lengths were taken to separate patients into two cohorts (with and without comorbidities) to better assess whether delay in growth or development was due to those factors, no appreciable difference was noted between the groups. Remarkably, without dividing the participants into two cohorts, there was not a statistically significant difference in milestones or growth at four years of age. This suggests these patients “catch up” developmentally. Explanations and validation of the ability to “catch up” in the face of seemingly growth-affecting comorbidities is beyond the scope of this paper.

Our data show hGH levels are not affected by propranolol therapy, but there are many factors other than hGH involved in pediatric growth that were not measured. Most children undergoing systemic propranolol treatment for IH did not have growth alterations. We were surprised to find some patients treated with propranolol had a higher weight-for-length BMI compared to WHO normative data. Although lengths and weights were within the 95^th^ percentile, the average BMI was higher. The initial concern of growth impairment is diminished; however, this may introduce a paradoxical concern of abnormal weight gain.

Care must be taken not to interpret this observation as a direct result of propranolol therapy. There is a population bias as this is a single-center study, and there is no control group of patients with hemangiomas not receiving therapy with whom to compare. There may also be a selection bias in the population of patients that presented to the center. These issues warrant further investigation. There are reports of patients taking propranolol for migraine that have experienced weight gain [32, 33]. However, this issue has not been studied in the pediatric population except for one study showing weight gain in a patient on prolonged propranolol therapy for 6-12 months [34].

As propranolol is a relatively new therapy for infantile hemangiomas, long-term studies are still ongoing to completely evaluate the effects of propranolol in the pediatric population. This study does not evaluate other reported side effects of propranolol such as hypoglycemia, insomnia, and bradycardia; therefore, it is not a comprehensive study of propranolol safety. Furthermore, the primary aim of this study was not to examine the efficacy of propranolol for hemangiomas, which have been previously shown in other studies [3, 9, 35] but instead to report the findings of the relationship between propranolol use, growth, and development.

Because therapy is effective and complete for most patients by one year of age, most patients do not follow in our clinic after the involution of the lesion for the 12 to 48-month period of milestone assessment. Thus, the questionnaire regarding milestone achievement after age one was completed by phone retrospectively. This methodology introduced recall bias.

## 5. Conclusion

We conclude that propranolol does not cause growth or developmental delay. This supports existing literature showing its safety in pediatric patients for the treatment of infantile hemangioma. Further study is warranted to evaluate the role of propranolol in the increase in weight-for-age and weight-for-length.

## Figures and Tables

**Table 1:** Patient characteristics.

Gender		
	Male	48
	Female	137
Birth history		
Premature		45
Twin or triplet		14
NICU admission		32
	Median NICU length of stay (days)	12
	Average NICU length of stay (days)	21
Location of IH		
	Head and neck	130
	Trunk	8
	Extremity	15
	Multifocal	31
	Unknown	1
Comorbidities that may affect growth or development		
	Yes	43
	No	142
	Cardiac abnormalities	16
	Laryngomalacia	5
	Non-hemangioma surgery	23
Gestational history (mother)		
	Preeclampsia	23
	Gestational diabetes	11
	Placental abnormalities	12
	First trimester bleeding	15
	Trauma	23
	Hypertension	23
	Tobacco or alcohol	8
	Amniocentesis	3
	Chorionic villus sampling	0
Other medications		
	None	53
	Antacid (PPI or H2 blocker)	61
	Systemic steroid	5
	Other	59

**Table 2:** Guardian reported side effects.

Side effect	Patients (% of total)
None	115 (62)
Reflux	27 (15)
Spit up more	19 (10)
Respiratory problems	13 (7)
Lethargy	9 (5)
Sleep disturbance	8 (4)
Mood alteration	6 (3)
Sweating	2 (1)
“Sickly”	2 (1)
Hyperactivity	2 (1)
Polyuria	1(<1)
Allergy	1(<1)
Gastritis	1(<1)

**Table 3:** Efficacy, side effects, and adjuvant therapy of propranolol based on guardian survey. Guardians may not have responded to each survey question thus creating variability in the number of respondents per question.

Efficacy		
Did not notice improvement		10
Time to notice an improvement	Less than one week	34
	Less than one month	32
	1-2 months	16
	More than 2 months	13
		95

Color change		
160	Yes	121 (76)
	No	39 (24)

Decreased size		
	Yes	158 (88)
180	No	22 (22)
		

Overall satisfaction		
120	Happy	94 (78)
	Neutral	16 (13)
	Unhappy	10 (8)

Side effects		
180	Yes	62 (34)
	No	118 (66)
		

Recurrence/rebound		
127	Yes	17 (13)
	No	110 (87)

Adjuvant treatments^∗^		
180	Laser	97 (54)
	Surgery	73 (41)
	Steroid	35 (19)
	Scar revision	1 (<1)
	Vincristine	3 (1)

^∗^Note: some patients had multiple treatments.

## Data Availability

Data is available upon request. Please contact gtrichter@uams.edu if more information is required.

## Supplementary Materials

Supplementary Materials Supplementary tables were included to provide the reader with further information regarding the number of children above and below WHO *z*-scores, as well as the relationship between anthropometric measurements and the length of propranolol therapy.
