# Supplementary material for: Propranolol for Treatment of Infantile Hemangioma: Efficacy and Effect on Pediatric Growth and Development
Source: Int J Pediatr. 2021 Apr 7;2021:6669383. doi: 10.1155/2021/6669383 (PMC8049833; doi:10.1155/2021/6669383)
Supplement: Supplementary Materials — Supplementary tables were included to provide the reader with further information regarding the number of children above and below WHO z-scores, as well as the relationship between anthropometric measurements and the length of propranolol therapy. [file 6669383.f1.docx]

**Supplementary Table 1: Number of children below/above WHO Z-scores – Excluding comorbidities:**

| **Age (months)** | **Weight-for-age z-score** | | | | **Total** |
| --- | --- | --- | --- | --- | --- |
|  | **<-2** | **<-1** | **>1** | **>2** |  |
| **<10** | 7 (4.6%) | 23 (15.2%) | 24 (15.9%) | 4 (2.6%) | 151 |
| **10-20** | 3 (2.2%) | 8 (5.9%) | 39 (28.9%) | 14 (10.4%) | 135 |
| **20-30** | 0 (0.0%) | 2 (3.5%) | 21 (36.8%) | 5 (8.8%) | 57 |
| **30-40** | 0 (0.0%) | 1 (2.8%) | 11 (30.6%) | 3 (8.3%) | 36 |
| **40-50** | 0 (0.0%) | 2 (6.5%) | 11 (35.5%) | 3 (9.7%) | 31 |
| **50-60** | 0 (0.0%) | 1 (6.7%) | 5 (33.3%) | 3 (20.0%) | 15 |
| **60-70** | 0 (0.0%) | 0 (0.0%) | 1 (20.0%) | 0 (0.0%) | 5 |
| **>80** | 0 (0.0%) | 0 (0.0%) | 0 (0.0%) | 0 (0.0%) | 1 |

| **Age (months)** | **Length/height-for-age z-score** | | | | **Total** |
| --- | --- | --- | --- | --- | --- |
|  | **<-2** | **<-1** | **>1** | **>2** |  |
| **<10** | 3 (3.1%) | 14 (14.6%) | 14 (14.6%) | 3 (3.1%) | 96 |
| **10-20** | 3 (3.3%) | 18 (20.0%) | 19 (21.1%) | 4 (4.4%) | 90 |
| **20-30** | 2 (3.8%) | 9 (17.3%) | 6 (11.5%) | 2 (3.8%) | 52 |
| **30-40** | 4 (11.4%) | 7 (20.0%) | 4 (11.4%) | 0 (0.0%) | 35 |
| **40-50** | 0 (0.0%) | 2 (7.7%) | 6 (23.1%) | 0 (0.0%) | 26 |
| **50-60** | 1 (9.1%) | 2 (18.2%) | 1 (9.1%) | 0 (0.0%) | 11 |
| **60-70** | 0 (0.0%) | 1 (20.0%) | 0 (0.0%) | 0 (0.0%) | 5 |
| **>80** | 0 (0.0%) | 0 (0.0%) | 0 (0.0%) | 0 (0.0%) | 1 |

| **Age (months)** | **Weight-for-length/height z-score** | | | | **Total** |
| --- | --- | --- | --- | --- | --- |
|  | **<-2** | **<-1** | **>1** | **>2** |  |
| **<10** | 2 (2.1%) | 10 (10.5%) | 19 (20.0%) | 2 (2.1%) | 95 |
| **10-20** | 1 (1.2%) | 5 (6.0%) | 26 (31.0%) | 8 (9.5%) | 84 |
| **20-30** | 0 (0.0%) | 0 (0.0%) | 25 (58.1%) | 6 (14.0%) | 43 |
| **30-40** | 0 (0.0%) | 0 (0.0%) | 9 (34.6%) | 4 (15.4%) | 26 |
| **40-50** | 0 (0.0%) | 1 (4.8%) | 10 (47.6%) | 4 (19.0%) | 21 |
| **50-60** | 0 (0.0%) | 1 (9.1%) | 4 (36.4%) | 3 (27.3%) | 11 |

| **Age (months)** | **BMI-for-age z-score** | | | | **Total** |
| --- | --- | --- | --- | --- | --- |
|  | **<-2** | **<-1** | **>1** | **>2** |  |
| **<10** | 2 (2.1%) | 12 (12.6%) | 17 (17.9%) | 1 (1.1%) | 95 |
| **10-20** | 1 (1.2%) | 6 (7.1%) | 23 (27.4%) | 8 (9.5%) | 84 |
| **20-30** | 0 (0.0%) | 0 (0.0%) | 25 (58.1%) | 7 (16.3%) | 43 |
| **30-40** | 0 (0.0%) | 0 (0.0%) | 10 (38.5%) | 4 (15.4%) | 26 |
| **40-50** | 0 (0.0%) | 1 (4.8%) | 10 (47.6%) | 4 (19.0%) | 21 |
| **50-60** | 0 (0.0%) | 1 (9.1%) | 4 (36.4%) | 3 (27.3%) | 11 |
| **60-70** | 0 (0.0%) | 0 (0.0%) | 1 (20.0%) | 0 (0.0%) | 5 |
| **>80** | 0 (0.0%) | 0 (0.0%) | 0 (0.0%) | 0 (0.0%) | 1 |

**Supplementary Table 2: Relationship between anthropometric measurements and length of propranolol therapy (All Patients)**

|  | Weight-for-age z-score | | | | | | |
| --- | --- | --- | --- | --- | --- | --- | --- |
| Months on propranolol | Total | >1 |  | *p*-value* | >2 |  | *p*-value* |
| 0 | 171 | 23 | 13.45% | Reference | 4 | 2.34% | Reference |
| 1 | 136 | 22 | 16.18% | 0.289 | 8 | 5.88% | 0.125 |
| 2 | 133 | 23 | 17.29% | 0.388 | 4 | 3.01% | 1.000 |
| 3 | 124 | 28 | 22.58% | 0.008 | 5 | 4.03% | 0.500 |
| 4 | 114 | 24 | 21.05% | 0.065 | 7 | 6.14% | 0.250 |
| 5 | 116 | 30 | 25.86% | 0.001 | 8 | 6.90% | 0.031 |
| 6 | 97 | 25 | 25.77% | 0.013 | 10 | 10.31% | 0.031 |
| 7-12 | 117 | 22 | 18.80% | 0.001 | 8 | 6.84% | 0.063 |
| More than 12 | 88 | 29 | 32.95% | 0.000 | 11 | 12.50% | 0.008 |

* McNemar's exact p-value comparing each month to time 0 (reference)

|  | BMI-for-age z-score | | | | | | |
| --- | --- | --- | --- | --- | --- | --- | --- |
|  |  | | | | | | |
| Months on propranolol | Total | >1 |  | *p*-value* | >2 |  | *p*-value* |
| 0 | 80 | 7 | 8.75% | Reference | 0 | 0.00% | Reference |
| 1 | 62 | 13 | 20.97% | 0.180 | 3 | 4.84% | 0.250 |
| 2 | 50 | 12 | 24.00% | 0.021 | 1 | 2.00% | 1.000 |
| 3 | 45 | 10 | 22.22% | 0.070 | 1 | 2.22% | 1.000 |
| 4 | 52 | 13 | 25.00% | 0.289 | 4 | 7.69% | 0.250 |
| 5 | 46 | 12 | 26.09% | 0.031 | 5 | 10.87% | 0.125 |
| 6 | 41 | 14 | 34.15% | 0.039 | 4 | 9.76% | 0.125 |
| 7-12 | 71 | 19 | 26.76% | 0.057 | 9 | 12.68% | 0.016 |
| More than 12 | 77 | 33 | 42.86% | <0.001 | 13 | 16.88% | 0.008 |

* McNemar's exact p-value comparing each month to time 0 (reference)

|  | Length/height-for-age z-score | | | | | | |
| --- | --- | --- | --- | --- | --- | --- | --- |
| Months on propranolol | Total | >1 |  | *p*-value* | >2 |  | *p*-value* |
| 0 | 81 | 13 | 16.05% | Reference | 2 | 2.47% | Reference |
| 1 | 63 | 8 | 12.70% | 0.688 | 2 | 3.17% | 1.000 |
| 2 | 50 | 9 | 18.00% | 1.000 | 1 | 2.00% | 1.000 |
| 3 | 45 | 9 | 20.00% | 0.125 | 1 | 2.22% | 1.000 |
| 4 | 53 | 13 | 24.53% | 0.549 | 4 | 7.55% | 1.000 |
| 5 | 49 | 6 | 12.24% | 1.000 | 1 | 2.04% | 1.000 |
| 6 | 43 | 6 | 13.95% | 0.688 | 1 | 2.33% | 1.000 |
| 7-12 | 78 | 16 | 20.51% | 0.302 | 3 | 3.85% | 1.000 |
| More than 12 | 85 | 9 | 10.59% | 1.000 | 3 | 3.53% | 1.000 |

* McNemar's exact p-value comparing each month to time 0 (reference)
